# Countrywide natural experiment links built environment to physical activity

**DOI:** 10.1038/s41586-025-09321-3

**Published:** 2025-08-13

**Authors:** Tim Althoff, Boris Ivanovic, Abby C. King, Jennifer L. Hicks, Scott L. Delp, Jure Leskovec

**Affiliations:** 1 Allen School of Computer Science & Engineering, University of Washington, Seattle, WA USA; 2 NVIDIA Research, Santa Clara, CA USA; 3 Department of Epidemiology & Population Health, Stanford University School of Medicine, Stanford, CA USA; 4 Stanford Prevention Research Center, Department of Medicine, Stanford University School of Medicine, Stanford, CA USA; 5 Department of Bioengineering, Stanford University, Stanford, CA USA; 6 Department of Mechanical Engineering, Stanford University, Stanford, CA USA; 7 Department of Computer Science, Stanford University, Stanford, CA USA; 8 Chan Zuckerberg Biohub, San Francisco, CA USA

**Keywords:** Risk factors, Epidemiology

## Abstract

While physical activity is critical to human health, most people do not meet recommended guidelines^1,2^. Built environments that are more walkable have the potential to increase activity across the population^3–8^. However, previous studies on the built environment and physical activity have led to mixed findings, possibly due to methodological limitations such as small cohorts, over-reliance on self-reported measures and cross-sectional designs^5,7,9–11^. Here we address these limitations by leveraging a large US cohort of smartphone users (*N* = 2,112,288) to evaluate within-person longitudinal behaviour changes that occurred over 248,266 days of objectively measured physical activity across 7,447 relocations among 1,609 US cities. By analysing the results of this natural experiment, which exposed individuals to differing built environments, we find that increases (decreases) in walkability are associated with significant increases (decreases) in physical activity after relocation. For example, moving from a less walkable (25th percentile) city to a more walkable city (75th percentile) increased walking by 1,100 daily steps, on average. These changes hold across different genders, ages and body mass index values, and are sustained over 3 months. The added activity is predominantly composed of moderate-to-vigorous physical activity, which is linked to an array of associated health benefits^1^. Evidence against residential self-selection confounding is reported. Our findings provide robust evidence supporting the importance of the built environment in directly improving health-enhancing physical activity and offer potential guidance for public policy activities in this area.

## Abstract (web summary)

By analysing the smartphone data of 2,112,288 participants, in particular observing and comparing the activity of the same individual in two different environments, we find that increases in the walkability of environments result in increases in daily physical activity, providing evidence of the importance of the built environment on physical health.

## Main

A substantial number of people worldwide are physically inactive^4,12,13^ and therefore at risk for common and deadly non-communicable diseases such as cardiovascular disease, cancer and diabetes^13–15^. Meanwhile, urban environments worldwide have grown rapidly, with current estimates predicting that 6.7 billion people will be living in cities by 2050 (ref. ^16^). While the evidence base on the impacts of the design of urban environments on physical activity levels has grown, further information is needed on the putative causal impacts of diverse urban environments on key health behaviours such as physical activity^5,7,10,11,17,18^ and interactions between environmental and individual factors^19^. Specifically, current evidence has not been able to determine whether physical activity levels are directly influenced by the built environment or are mainly a product of personal preferences^10,20^. Understanding these factors is critical for developing optimal public policy^1,8,21^, and for planning cities^5,22^ and designing behaviour change interventions^23,24^.

Previous studies on the effects of the built environment on physical activity have led to mixed or modest findings and have not been able to reliably distinguish between direct environmental impacts and individual preferences. Common methodological limitations include small cohorts, data from a single location or a limited number of locations, over-reliance on self-reported activity, with its attendant biases^25^, cross-sectional designs that constrain temporal understanding and causal inference, residential self-selection and other confounding factors^5,7,9,20,26,27^. Today’s mobile phones, including the now globally dominant smartphone, can capture physical activity and geolocation in a continuous fashion, making them a powerful tool for studying large-scale population dynamics and health^23^, the use of which can reveal the basic patterns of physical activity^3^, sleep^28^, human movement^29^ and mood rhythms^30^, along with the dynamics of the spread of diseases such as malaria^31^ and COVID-19 (ref. ^32^) and linkages with socioeconomic status in low- and middle-income countries^33^. In this study, we use a large-scale physical activity dataset to disentangle the influences of the built environment from personal proclivities through a natural experiment, and quantify the impact of walkability on changes in physical activity levels at the individual and population scale.

## Effect of walkability on daily steps

**Fig. 1:**
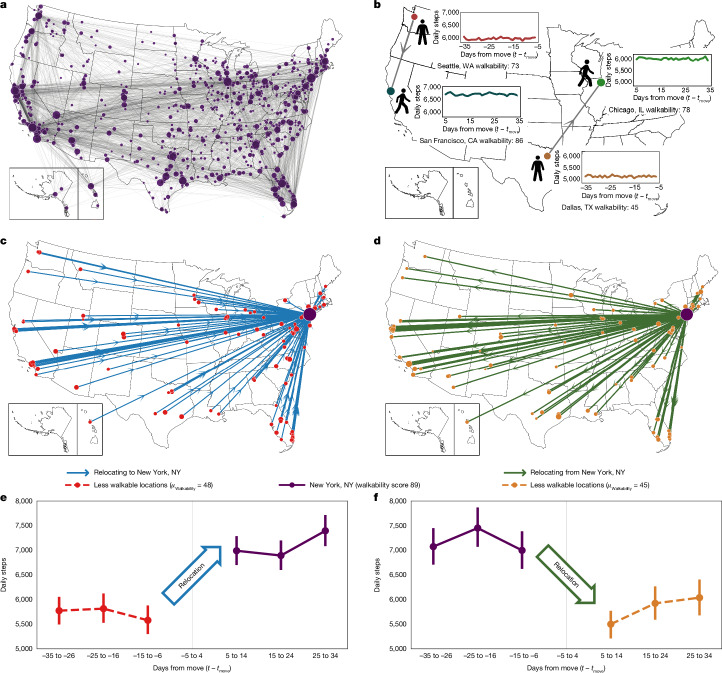
Physical activity levels undergo significant changes following relocation between US cities of different walkability levels. **a**, During the observation period, 5,424 participants relocated 7,447 times between 1,609 US cities. Circle area is proportional to the square root of the number of relocations to and from the city. **b**, The physical activity levels of participants were tracked through smartphone accelerometry over several months before and after relocation, creating a countrywide study of 7,447 quasi-experiments. **c**–**f**, Physical activity of participants moving from less walkable locations to New York City (**c**,**e**), in comparison to participants moving in the opposite direction (**d**,**f**) (Methods). Activity levels change significantly immediately after relocation and are symmetric but inverted for participants moving in the opposite direction (**e**,**f**). All error bars throughout figures correspond to bootstrapped 95% confidence intervals. Credits: **a**–**d**, maps reproduced from US Census Bureau (https://www.census.gov/geographies/mapping-files/2016/geo/carto-boundary-file.html); **b**, walking human silhouette reproduced from Wikimedia commons under a Creative Commons CC BY 1.0 license.

We study 248,266 days of minute-by-minute step recordings from 5,424 users of the Azumio Argus smartphone application who relocated at least once within a 3-year observation period. Overall, these participants relocated a total of 7,447 times among 1,609 cities within the USA, forming a countrywide natural experiment (Fig. 1a). The dataset includes smartphone-derived accelerometry recordings of physical activity for free-living individuals that were exposed to different built environments, enabling us to compare their objectively measured, longitudinal physical activity for up to 90 days before and after relocation (Fig. 1b). The average participant recorded 5,574 steps per day (standard deviation *σ* = 3,055) over an average span of 14.2 hours. Research has demonstrated that smartphones provide accurate step counts^34^ and reliable activity estimates in both laboratory and free-living settings^35^. We ensured that our dataset included a broad range of relocating and non-relocating participants, including across age, gender and weight status (as measured by body mass index (BMI)) (Extended Data Table 1). Previous work further verified that data from the Argus smartphone application used in this study reproduced established relationships between age, gender, weight status and activity, as well as country-level variations in activity and obesity levels^3^. We also verified that our findings were robust to different relocation definitions, including those aimed at removing business and leisure travel and excluding time periods right before and right after relocation, which are probably impacted by the relocation process itself (Extended Data Fig. 6; Methods: ‘Identifying participant relocation’).

Our large-scale activity measurements enable us to characterize the impact of built environments on physical activity. Consider the 178 participants relocating to New York City (a walkability score of 89 out of 100) coming from various less walkable US locations (Fig. 1c; a Walk Score of at least one standard deviation or 15.4 points lower; mean walkability 48). When exposed to the built environment of New York City after relocating, these participants increased their physical activity by 1,400 steps, their average daily steps increasing from 5,600 to 7,000 (Fig. 1e; *P* < 10^−10^; all statistical hypothesis tests throughout refer to two-sided Student’s *t*-tests unless indicated otherwise; Methods: ‘Statistical methods’). Participants relocating in the opposite direction, that is, from New York City to other less walkable US cities (Fig. 1d), exhibited an inverted, symmetric effect of decreasing their physical activity by 1,400 steps, going from 7,000 to 5,600 average daily steps (*P* < 10^−10^, Fig. 1f; more examples in Supplementary Fig. 1).

**Fig. 2:**
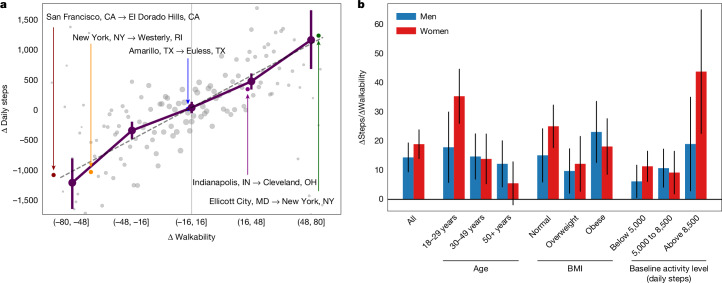
Relocations with changes in walkability are associated with corresponding changes in physical activity across most demographics. **a**, Difference in average daily steps aggregated across all relocations. We find that significantly more walkable locations are associated with increases of about 1,100 daily steps, and significantly less walkable locations are associated with similar decreases (for 49–80 point Walk Score increase or decrease). Moving to locations of similar walkability is associated with unchanged physical activity levels. **b**, Higher walkability is associated with increased daily steps across age, gender, BMI and baseline activity level groups. Bars show the steps gained per day for each point increase in walkability score (assuming linear model; Methods). Positive values across all bars reveal that, with increasing walkability, more steps are taken by every subgroup, which is significant for all the subgroups except women over 50 years of age (Student’s *t*-tests, all *P* < 0.05; women over 50 years of age, *P* = 0.14).

To investigate whether moving to more walkable environments generally leads to increased physical activity, we aggregate changes in physical activity across all relocations in the dataset (Fig. 2a; Methods: ‘Aggregating relocation-based quasi-experiments’). We find that relocations to more walkable cities (Walk Score increases of 49 and higher) are associated with increases of about 1,100 daily steps, equivalent to 11 minutes more walking activity every day^36^.

## Evaluating potential selection effects

Notably, we attempt to estimate the impact of substantial built environment changes in-place on physical activity through events of substantial built environment changes resulting from relocation. This approach provides unique advantages, as substantial in-place changes are exceedingly rare and costly, leading to recent calls for quasi-experimental study designs such as ours^37^. However, this approach could lead to biased estimates due to selection effects, such as participants moving to a new environment being particularly motivated to increase physical activity. Importantly, we find robust evidence that our estimates are unlikely to be significantly influenced by such selection effects. Critical to this argument are (1) that we find no evidence of increases in average physical activity of participants when they are relocating to environments with walkability scores similar to the environment from which they came (bootstrapped 95% confidence interval for walkability differences between −16 and 16 is [−76 to 122]) and (2) that the estimated relationship between walkability differences and physical activity is approximately point symmetric (Fig. 2a and Supplementary Fig. 2). If participants that moved were motivated to increase their physical activity after moving, we should have observed this increase also for relocations to environments of similar walkability, but we did not observe any difference. If participants that moved relocated to higher-walkability locations specifically for this quality, a form of residential self-selection, we should have observed higher physical increases relative to physical activity decreases when relocating to a lower-physical-activity location. Instead, we observed point symmetric changes.

In addition, we observe that these increases are sustained over 3 months after moving (Supplementary Fig. 3c and Extended Data Fig. 2). Furthermore, we find similar, consistent effects of walkability increases and decreases between cities in similar climates (for example, Ellicott City, MD to New York, NY in Fig. 2a), and more generally across relocations during all seasons (Supplementary Fig. 6), and after relocating to cities of higher, similar and lower median household income (Supplementary Fig. 7). In addition, census data suggest that between 77% and 98% of participants that move do not move for walkability reasons, but instead for family, job and housing-related reasons (Methods: ‘Selection effects in relocation and mobile app usage’). We also find that relocating and non-relocating app users are similar in age, gender and weight status, and that those relocating to higher-, similar- and lower-walkability locations were similar in age, weight status and previous physical activity levels (Extended Data Figs. 4 and 5; Methods: ‘Selection effects in relocation and mobile app usage’). Overall, these results suggest that physical activity levels are directly influenced by the built environment and not simply a product of personal preferences or other types of selection effects.

## Walkability effects across demographic groups

We find that higher walkability is associated with significantly more daily steps across all age, gender, BMI and baseline activity level groups, which is significant for all the subgroups except women over 50 years of age (Fig. 2b; all *P* < 0.05; women over 50 years of age, *P* = 0.14). Previous research has identified additional barriers to physical activity relevant to older women including cultural expectations, norms, societal messages discouraging physical activity, family priorities and safety^38,39^. The relationship between walkability and activity is strongest for highly active women (gaining 43.7 steps per walkability point increase). Importantly, we find that regardless of BMI status, individuals record more steps after moving to more walkable cities, and that these increases are also shared by individuals who were less active before moving (Fig. 2b). These findings suggest that compared with interventions targeting individuals and reaching small numbers of people, changes to the built environment can influence large populations. However, the relatively smaller effect for older women suggests that, for this group in particular, built environment changes may need to be accompanied by additional age- and gender-specific interventions aimed at their specific constraints. Previous work has described person-level factors that impede physical activity participation of older women, such as a greater number of functional impairments that discourage activity, more frequent caregiving demands that interfere with physical activity, more difficulties with outdoor wayfinding and lower driving rates, which can limit their ability to get to local parks to exercise^40–45^. In addition, older women are less well served by public transit in many US cities, which base their routes on commuter patterns^46,47^. Therefore, multilevel interventions that focus on social environmental factors in addition to built environment factors are recommended, such as encouraging walking groups and advising on how to overcome the above types of personal barriers^41,48^.

## Changes in moderate-to-vigorous physical activity

**Fig. 3:**
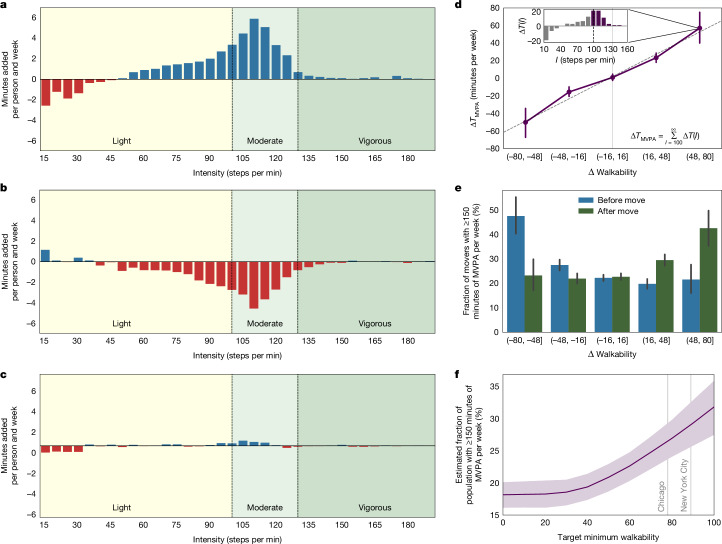
Improvements in walkability are associated with increases in MVPA and with twice as many participants meeting aerobic physical activity guidelines (49+ point increase). **a–c**, Changes in physical activity stratified by intensity of physical activity (steps per minute) following relocation to more (**a**; more than 16-point walkability increase), less (**b**; more than 16-point walkability decrease) and similarly (**c**; 16-point walkability difference or less) walkable environments. **a**, We find that walkability-induced additional physical activity (Fig. 2a) predominantly consists of MVPA, which has been shown to be beneficial for many health outcomes^14,15^. **b**, Moving to less walkable locations is associated with a symmetric loss of MVPA that is equivalent to the increase in more walkable locations (**a**). **c**, Further, moving to similarly walkable locations is associated with an unchanged distribution of intensity levels. This suggests that relocation, in and of itself, is not generally associated with increases in physical activity, for instance, owing to an individual’s motivation to increase physical activity. **d**, Change in MVPA (minutes per week) versus differences in walkability. Δ*T*(*I*) is defined as the change in weekly minutes (Δ*T*) of activity at intensity level *I* after relocation, in units of steps per minute. Δ*T*_MVPA_, that is, change in the time spent in MVPA, is computed by summing Δ*T*(*I*) for *I* ≥ 100 (inset). Large increases in walkability (that is, 49–80 points) are associated with an increase of about 1 hour per week in MVPA. **e**, The increases in time spent in MVPA lead to twice as many participants meeting national and international aerobic physical activity guidelines of 150 minutes per week or more in MVPA (before 21.5%, after 42.5%). **f**, A simulation based on these estimates predicts that if all US cities had the walkability of Chicago or Philadelphia (a walkability score of 78), then individuals would increase their average daily step activity by 443 steps and their MVPA per week by 24 minutes, which would mean that 11.2% or 36 million more Americans would then meet national physical activity guidelines for MVPA (Methods).

Next, we investigate whether the walkability-induced increase in steps reflected an increase in moderate-to-vigorous physical activity (MVPA), which has been shown to be beneficial for many health outcomes, including lower all-cause mortality risk^14,15^. Using minute-by-minute step data, we find that extra steps taken after moving to a more walkable location are predominantly composed of MVPA corresponding to brisk walks (Fig. 3a). We estimate that large increases in walkability (that is, 49–80 points) are associated with an increase in MVPA of about 1 hour per week (Fig. 3d). Further emphasizing the consistency and symmetry of built environment effects, we find that similar amounts of MVPA are lost when relocating to a less walkable location (Fig. 3b), and that the activity intensity distribution remains effectively unchanged when relocating to a similarly walkable location (Fig. 3c). US national physical activity guidelines recommend, similar to international guidelines, 150 minutes or more per week of MVPA to obtain optimal health benefits^49^. For a walkability increase of between 48 and 80 points, we find that the associated increases in MVPA would support 42.5% of participants meeting guidelines for MVPA versus 21.5% before relocation, a 98% relative increase (Fig. 3e; Methods: ‘Physical activity measure’ and ‘Simulating the impact of walkability improvements’). Our findings substantively expand on the findings of previous literature, indicating that improving the walkability of built environments can lead to better health outcomes across large populations.

We perform a simulation study to predict how improving walkability would support increasing the fraction of the US population that meets aerobic physical activity guidelines (Methods: ‘Simulating the impact of walkability improvements’). Our dataset covers 1,609 US cities, which are home to more than 41% of the country’s population (137 million), and we adjust for age differences between the smartphone user population and the US adult population. According to our smartphone-based objective measurements, about 18% or 58 million Americans met the guidelines for MVPA between 2013 and 2016. Our estimate of 18% meeting aerobic guidelines is within expectations, given well-established differences between accelerometer-derived and self-reported physical activity^50,51^. Our simulation (Fig. 3f) predicts that bringing all US locations to the level of Chicago or Philadelphia (a walkability score of 78) may lead to 11.2% or 36 million more Americans meeting aerobic physical activity guidelines. Bringing all US locations to the level of New York City (a walkability score of 89) may lead to 14.5% or 47 million more Americans meeting these guidelines.

## Discussion

There are limitations to the device-based instrument (that is, people’s personal smartphones) we used to collect physical activity data in participants’ natural environments. For example, our sample may be biased towards individuals of higher socioeconomic status and people interested in their activity and health. However, we find that walkability improvements led to increased physical activity after relocating to cities of higher, similar and lower median household income (Supplementary Fig. 7). We further acknowledge that other city characteristics may affect walking and be correlated with the city’s walkability (for example, climate, availability of transit, or land use mix)^52,53^. However, we find that walkability differences are associated with physical activity differences in cities of similar climate (Supplementary Fig. 5) and across all seasons (Supplementary Fig. 6). While relocation uniquely enables the quasi-experimental study of behavioural changes in different environments, there may be selection effects driving relocation, referred to as residential self-selection^20^. However, as previously discussed, we report evidence against such selection effects.

Over 90% of adults in the USA already own a smartphone^54^ and the number of mobile connections worldwide has risen to 8.5 billion^55^, exhibiting significant year-to-year increases. Therefore, we expect any biases related to smartphone ownership and usage to continue to diminish in the future. This study is restricted to a single country and results may not generalize to other countries. However, previous studies have found, in general, similar types of built environment relationships across countries diverse in climate, demographics, income, culture and activity supportiveness^5,56–58^. As these studies used walkability indices that were based on elements shared with the measure used here (Methods: ‘Walkability measure’), this suggests that our findings may generalize to other countries. We chose a simple, highly used and extensively validated measure of walkability at a city level^59–64^. However, this type of aggregated, non-divisible walkability score precludes the ability to identify which elements of walkability may confer the largest benefits. Location data to assess walkability on a neighbourhood or census tract level was not available, preventing the analysis of within-city variation in walkability. Further research is needed to identify key environmental features on a neighbourhood level and disentangle their individual contributions, building on past cross-sectional research and smaller-scale studies using self-reported physical activity measures, which currently constitute the majority of research in the field^65^. Future research is also needed to untangle how the built environment may differentially affect physical activity related to leisure, transportation and working. While walking is the most popular aerobic physical activity^66^, our dataset may fail to capture time spent in activities where it is impractical to carry a phone (for example, football) or for which steps are not a major component of the activity (for example, bicycling), and there may exist systematic differences in wear time because participants in the current dataset had to carry their phone for steps to be recorded. The increasing prevalence of wearable activity trackers in the form of smartwatches and similar devices will continue to enable more convenient methods of capturing daily movement and steps. Further, our smartphone dataset reproduces previously established relationships between activity across geographic locations, gender and age^3^. We also find that the span of time over which steps were recorded is uncorrelated with relocating to higher- or lower-walkability areas (Extended Data Fig. 7), and thus systematic wear time differences are unlikely to affect our analyses. Together, these findings increase confidence that our dataset is able to identify activity differences between built environments and groups based on gender, age and weight status.

Further research is needed to determine which policies, on topics such as land use mix, intersection design and access to public transit, are most effective in increasing city walkability for both new and existing urban areas^53^. While increasing walkability of all cities to the level of New York City is probably not possible, earlier research has shown promising connections between implemented changes to the built environment, walkability and physical activity^67^. The results of our analysis will provide researchers and policy-makers with the information to estimate the effects of targeted increases in walkability on physical activity and weigh the cost effectiveness of changes to the built environment against other public health interventions.

This countrywide natural experiment presents prospective evidence of built environments affecting physical activity across 7,447 relocations among 1,609 US cities over a 3-year timespan. It reveals the direct behavioural impacts of differing built environments on the physical activity levels of individuals and demonstrates the utility of such massive, digitally enabled, real-world datasets for evidence-based policy. Our findings suggest that designing built environments to be more activity-friendly could have significant effects on the physical activity of large populations, and serve as a powerful complement to interventions that focus on changing behaviour at the individual level. However, changes in built environments may need to be accompanied by additional age- and gender-specific interventions aimed at specific subgroups who could particularly benefit from physical activity increases (that is, women over 50 years old). The quality of the prospective device-collected evidence and consistency of findings across numerous cities, demographic groups and relocation-related walkability differentials highlight the fundamental importance of the urban built environment in improving physical activity and health.

## Methods

### Study design

We conducted a countrywide, prospective, longitudinal physical activity study of US residents that evaluated their physical activity levels within the context of the walkability of their built environments before and after relocation (‘participants’). We leveraged the naturally occurring physical activity data that was captured by a health app on participants’ phones to compare each person’s physical activity levels before and after they relocated to a different area within the USA. While similar relocation-based study designs have been used previously to estimate effects of place and built environments^26,68,69^, the vast majority have been limited by relatively small sample sizes, using only self-report physical activity measurement and the limited diversity with respect to the areas to which they relocated. Objective measures of both urban walkability and physical activity were used and are discussed in more detail throughout the Methods. We analysed anonymized, prospectively collected data from 2,112,288 US smartphone users using the Azumio Argus health app over 3 years (March 2013 to February 2016) to identify 5,424 participants that relocated 7,447 times among 1,609 US cities. These 1,609 cities are home to 137 million Americans, or more than 42% of the US population. We note that relocation is neither purely exogenous nor random, and discuss the important implications of this below. We follow established best practices for analysing large-scale health data from wearables and smartphone apps^70^.

The Azumio Argus app is a free smartphone application for tracking physical activity and other health behaviours. Participants were excluded from a particular analysis if necessary information was unreported (for example, participants with no reported age were excluded from the analysis of Fig. 2b). Extended Data Table 1 includes basic statistics on study population demographics and weight status (BMI). Anonymized Azumio Argus app data was obtained through a Data Use Agreement. Data handling and analysis was conducted in accordance with the guidelines of the Stanford University Institutional Review Board, which deemed this study exempt.

For population size statistics, refer to Extended Data Tables 1–3.

### Statistical methods

All error bars throughout this paper correspond to bootstrapped 95% confidence intervals. When these bootstrapped 95% confidence intervals do not include the null value (typically 0), they indicate a statistically significant difference at the *α* = 0.05 level. All statistical hypothesis tests were two-sided Student’s *t*-tests unless indicated otherwise.

### Identifying participant relocation

We defined participant relocation as the action of moving to a new place for a substantial amount of time. We identified participant relocation as follows. Participant location on a given day was assigned to a city based on the weather update in the participant’s app activity feed. Weather updates are automatically added to the feed of each participant according to the nearest cell phone tower. We searched for participants that stayed in one location within a 100-km radius for at least 14 days and then moved to a different location that was at least 100 km away. Participants were required to stay within a 100-km radius of this new location for at least another 14 days. The 14-day threshold was chosen to filter out short trips that may be related to business or leisure travel. Using this threshold, we find that most participants do not relocate again and spend a median of 81 days in the new location, effectively excluding the impact of short-term travel on our analyses. Most participants stopped tracking their activity at this time, rather than relocating again. In addition, we repeated our analyses with thresholds of 21 and 30 days and found highly consistent results (Extended Data Fig. 6). We required a substantial move distance (100 km or more) to ensure that relocating participants were exposed to a new built environment. We allowed for up to 5 days of intermediate travel between these two locations and ignored these days during analyses. We applied this method to 2,112,288 users of the Argus smartphone app and identified 31,034 relocations. Among these, we required participants to have used the app to track their physical activity for at least 10 days within the 30 days before and after their relocation (as in previous work^3^). We further required at least 1 day of tracked physical activity before and after relocation to ensure that, whenever we compare two participant populations, these populations are identical and therefore comparable (that is, we seek to identify within-participant changes in physical activity). We repeated our statistical analyses with alternative data inclusion criteria, such as the number of days with tracked physical activity, and found similar results.

### Physical activity measure

Our device-based (historically often called objective) measure of physical activity was the number of steps over time recorded by the participant’s smartphone. Steps were determined based on the smartphone accelerometers and the manufacturer’s proprietary algorithms for step counting. The Azumio Argus app records step measurements on a minute-by-minute basis. These measurements are collected passively without requiring the smartphone or Azumio Argus app to be in active use. Extended Data Table 2 includes basic statistics on physical activity and tracking in the study population.

Data from the Azumio Argus app have been used previously to study physical activity in large populations^3,71,72^, where the authors showed that this form of data follows well-established trends^3^. For example, they demonstrated that activity decreased with increasing age^12,19,73,74^ and BMI^19,74,75^, and is lower in female individuals than in male individuals^12,19,73,74,76^, trends that are consistent with national surveillance data in this area. Physical activity estimates were also reasonably well correlated with self-report-based population estimates on a country level^3^.

Several studies have established significant differences between accelerometer-derived and self-reported physical activity^50,51^. Self-reports typically overestimate moderate and vigorous activity and underestimate sedentary activity^50^. In a US study using National Health and Nutrition Examination Survey 2005–2006 data, 59.6% of adults self-reported meeting MVPA guidelines for aerobic physical activity, whereas estimates using accelerometry were much lower at 9.6%^51^. For our observation period between 2013 and 2016, the US National Health Interview Survey reported that 49.6–52.6% of the US population met MVPA guidelines. Nationally representative accelerometer-based estimates for this time are not available. Our smartphone-accelerometry-based estimate of 18% meeting aerobic guidelines is within expectations, given well-established differences between accelerometer-derived and self-reported physical activity and earlier data (Methods)^50,51^. In addition, unlike many previous studies mailing accelerometers to study participants to wear for a week, our study focuses on real-world physical activity by free-living individuals that may not be equally affected by their awareness of being observed (that is, the Hawthorne effect).

We filtered out days as invalid when less than 500 or more than 50,000 steps had been recorded. We further ignored days immediately preceding and following the relocation itself (5 days before and 5 days after relocation), because the process of relocating, rather than the new built environment itself, could impact physical activity during these days. Physical activity was relatively stable outside this period (Supplementary Fig. 4). We considered physical activity within a window of 30 days before and 30 days after relocation (with the exception of Supplementary Fig. 3 and Extended Data Fig. 2 that use 90-day windows to illustrate long-term changes). In total, our dataset included 248,266 days of objectively measured minute-by-minute physical activity surrounding 7,447 relocations (595,803 days for the 180-day period).

We used the following measures as primary outcomes in this study: (1) Change in average daily steps following relocation (Figs. 1e,f and 2a,b). (2) Change in average weekly minutes spent in MVPA following relocation, where we considered all minutes spent at intensities greater than or equal to 100 steps per minute as MVPA^36^: $\Delta {T}_{{\rm{MVPA}}}={\sum }_{I=100}^{\infty }\Delta T(I)$, where Δ*T*(*I*) is defined as the change in weekly minutes of activity at intensity level *I*, in units of steps per minute, after moving. Figure 3a–c shows changes in average weekly minutes spent at different intensity levels. (3) Change in the fraction of the population that met aerobic physical activity guidelines following relocation, defined as spending at least 150 minutes per week in MVPA^1^ (Fig. 3e,f). All error bars correspond to bootstrapped 95% confidence intervals^77^.

### Walkability measure

We considered relocations among 1,609 cities in the USA. Walkability scores for these cities were based on the publicly available and systematically developed Walk Score^78^. Scores are on a scale of 1 to 100 (where 100 is the most walkable) and are based on amenities (for example, grocery stores, schools, parks, restaurants and retail) within a 0.25-mile to 1.5-mile radius (a decay function penalizes more distant amenities) and measures of friendliness to pedestrians, such as city block length and intersection density. Extended Data Table 3 includes basic statistics on the cities included in our study and their walkability scores. Walk Scores at the city level are computed by weighting the Walk Score of each geographic unit within a city (typically about the size of a city block) by the population density of that unit^79^.

The Walk Score measure is a frequently used measure of walkability that is freely and widely available across the USA and other countries including Canada and Australia^78^. It is highly correlated^62^ with other walkability measures^80–82^, and was found to offer the best fit to walking trips in a study conducted in Montréal^62^. It is widely used in the literature and has been extensively validated^59–64^. Although other measures of walkability exist^80–82^, the Walk Score measure was chosen in light of the pragmatic focus of the investigation and its ease of use and accessibility. More comprehensive walkability indices could provide further granular information related to specific aspects of walkability that might be of prime importance.

We determined cut points for Walk Score differences of −16 to +16, 16 to 48 and 49 to 80, as we preferred cut points that were symmetric around 0 (no change in walkability score), equivalent in size (32 Walk Score points difference) and balanced granularity and statistical power, as large Walk Score differences are more rare. Among the 7,447 relocations, 2.4% (2.4%) were associated with 49+ walkability point increases (decreases), 20.7% (21.3%) were associated with 16–48 walkability point increases (decreases) and 53.1% of relocations were to locations of similar walkability (−16 to +16 point difference).

### Aggregating relocation-based quasi-experiments

We aggregated changes in physical activity following relocation based on the difference in walkability scores between the origin and destination city, Δ. In Fig. 2a, each circle corresponds to a pair of cities sized by the number of participants moving between those cities. We fit a linear model *m*Δ + *b* to these data with slope *m* = 16.6 (Student’s *t*-test; *P* < 10^−10^) and intercept *b* = 25.0 (Student’s *t*-test; *P* = 0.462).

We considered potential confounders such as differences in climate (using Köppen climate type^83^) and median income between the origin and destination city. We found that the relationship between walkability and walking behaviour still holds within pairs of cities with similar climate, for instance, moving from Miami, FL to Jacksonville, FL, or from Amarillo, TX to Euless, TX (see annotations in Fig. 2a as well as more generally in Supplementary Fig. 5). Furthermore, we found similar effects across relocations in all seasons (Supplementary Fig. 6) and relocations to cities with higher, lower and similar median household income levels (Supplementary Fig. 7).

### Impact of walkability across subgroups

We considered the effect of walkability differences on change in physical activity across subgroups based on demographics (ages 18–29, 30–49 and 50+ years), weight status (normal, overweight and obese levels of BMI), previous activity level (below 5,000, 5,000–8,500 and above 8,500 average daily steps before relocation) and gender (men and women). Owing to the approximately linear nature of the relationship between walkability changes and physical activity changes (Fig. 2a), we used a linear model for estimation. For each subgroup, we ran independent linear regressions of the difference in daily steps on differences in walkability between cities at the level of individual relocations. The models included an intercept coefficient: *m* ⋅ Δ + *b*. We determined the estimated coefficient of walkability (*m*; that is, the increase in daily steps for each one-point increase in walkability of a city) along with 95% confidence intervals (based on Student’s *t*-distribution) for each subgroup (Fig. 2b). We performed Student’s *t*-tests on the regression model coefficients, which establish that relocation to a city of higher walkability is associated with significantly more daily steps across all age, gender, BMI and activity level groups (Student’s *t*-test; all *P* < 0.05), with the exception of women over 50 years old, for which the positive difference was not statistically significant (Student’s *t*-test, *P* = 0.14). We found that the effect was diminished in overweight and obese women relative to normal-weight women. Thus, the non-significant effect on women over 50 years of age may be explained, in part, by the larger average BMI of this group (27.4) compared with other women (25.3; *P* < 10^−10^). In comparison, men over 50 years of age also had a larger BMI compared with other men, but the difference was smaller than in women (28.2 versus 27.0; *P* < 10^−7^).

### Adjusting for seasonality

Physical activity is influenced by climate and weather^84^ and relocations are not equally distributed across seasons (Supplementary Fig. 3a). We found that differences in physical activity levels following relocations may be influenced by seasonal variation, especially when considering comparatively long observation periods of about 6 months (Supplementary Fig. 3b,c). For analyses of variation in activity over time (Fig. 1e,f, Extended Data Fig. 2 and Supplementary Figs. 1 and 3), we adjusted for these seasonal effects by weighting relocations in each calendar month equally. This was achieved by first estimating physical activity levels separately for each calendar month and then taking the average. This process is repeated 1,000 times in our bootstrap estimates.

### Selection effects in relocation and mobile app usage

**Extended Data Fig. 1:**
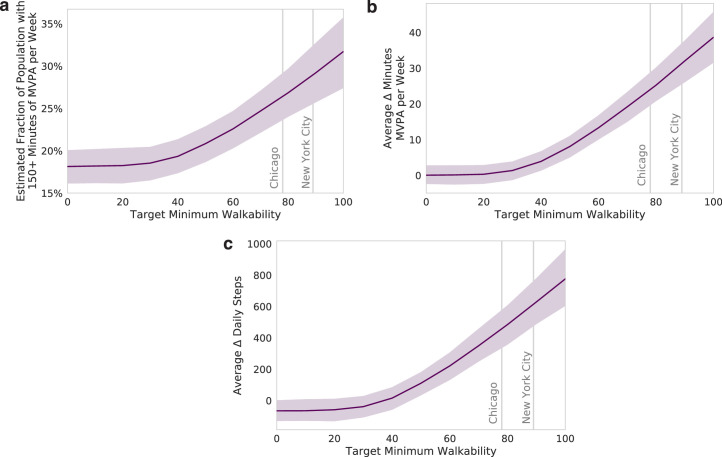
Simulating the impact of walkability improvements on United States physical activity levels. **a**, Estimated fraction of population with 150 minutes or MVPA or more per week following an increase in walkability across all represented U.S. locations. **b**, Average amount of MVPA added across population following an increase in walkability across all represented U.S. locations. **c**, Average amount of daily steps added across population following an increase in walkability across all represented U.S. locations. Shaded regions correspond to 95% confidence intervals.

**Extended Data Fig. 2:**
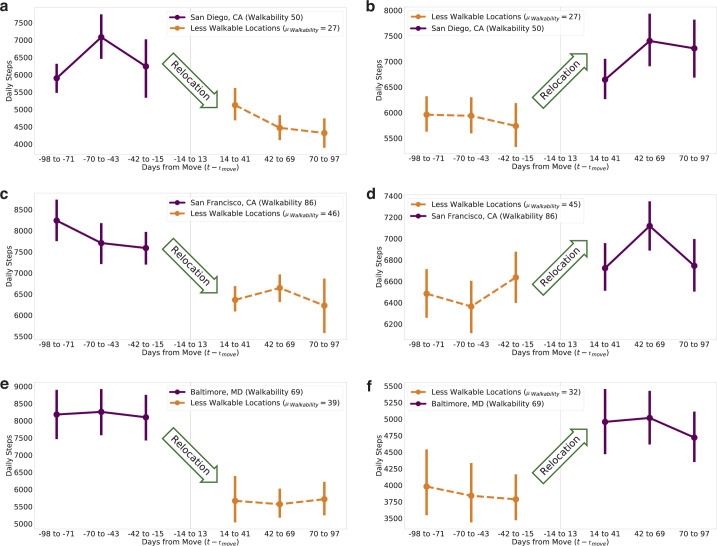
Changes in physical activity levels following relocation are still observed after three months. Examples show physical activity levels for participants moving from/to San Diego, CA (**a,b**), San Francisco, CA (**c,d**), and Baltimore, MD (**e,f**). The observation period is extended from 30 days to 90 days pre- and post-relocation. Observed changes in physical activity levels suggest that built environment influences persist over at least three months and may lead to sustained long-term behavior change.

**Extended Data Fig. 3:**
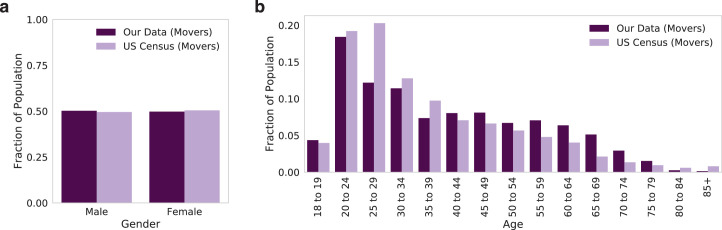
Demographics of relocating participants (Movers) in our dataset compared to U.S. Census estimates for movers. **a**, While there are no significant differences in the gender distribution (49.8% female vs. 50.4% female, *P* = 0.423, Z-test), **b**, We find a slightly lower age in our data for movers compared to the US Census reported movers (36.0 vs 37.7 median age), that we corrected for in the simulation experiment (Fig. 3f, Extended Data Fig. 1).

**Extended Data Fig. 4:**
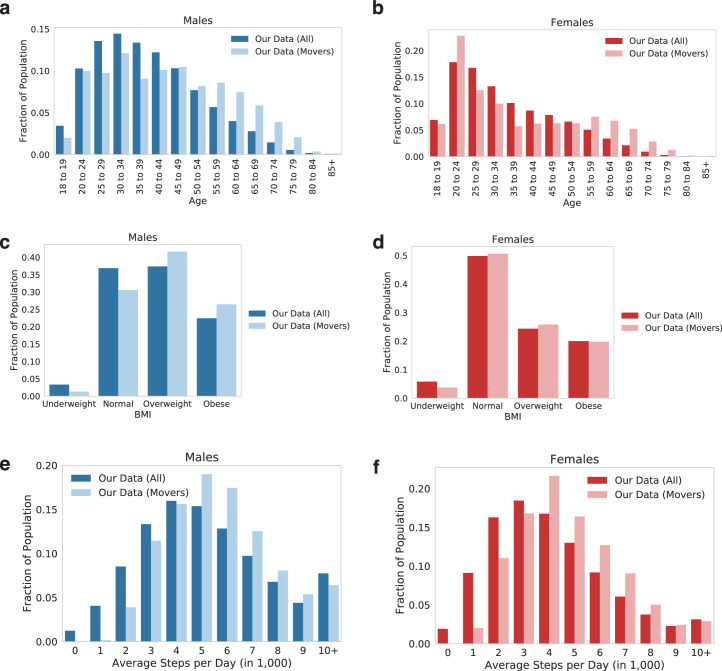
Comparison of relocating participants (movers) to all participants in our dataset to understand potential selection effects. Within the study population, we find that movers and non-movers (*i.e*., relocating and non-relocating participants) tend to be **a-b**, close in age (43.8 vs 37.9 and 38.5 vs 33.7 average age for men and women, respectively), **c-d**, and weight status (68.1 vs 59.8 and 45.6 vs 44.3 percent overweight and obese for men and women, respectively). **e-f**, However, movers were generally more physically active than non-movers (6,284 vs 5,825 and 5,279 vs 4,635 average daily steps for men and women, respectively).

**Extended Data Fig. 5:**
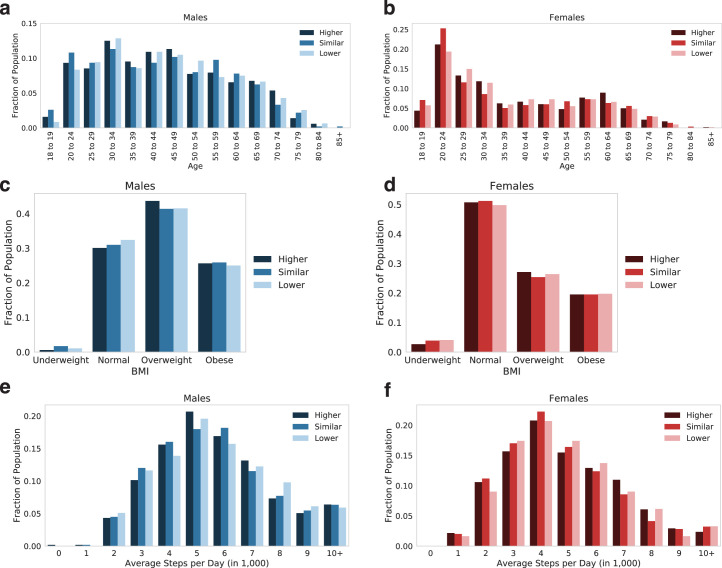
Comparison of different groups of relocating participants (movers) to understand potential selection effects. Within the study population, we find that movers to higher (greater than 16 walkability points), similar (within 16 walkability points), and lower (less than -16 walkability points) walkability locations tend to be **a-b**, close in age (44.4 vs 43.7 vs 45.0 and 39.0 vs 38.3 vs 38.3 average age for men and women moving to higher, similar, and lower walkability locations, respectively), **c-d**, weight status (69.3 vs 67.3 vs 66.5 and 46.6 vs 44.9 vs 46.1 percent overweight and obese for men and women moving to higher, similar, and lower walkability locations, respectively), **e-f**, and baseline physical activity levels (6,257 vs 6,229 vs 6,301 and 5,422 vs 5,239 vs 5,405 average daily steps for men and women moving to higher, similar, and lower walkability locations, respectively).

**Extended Data Fig. 6:**
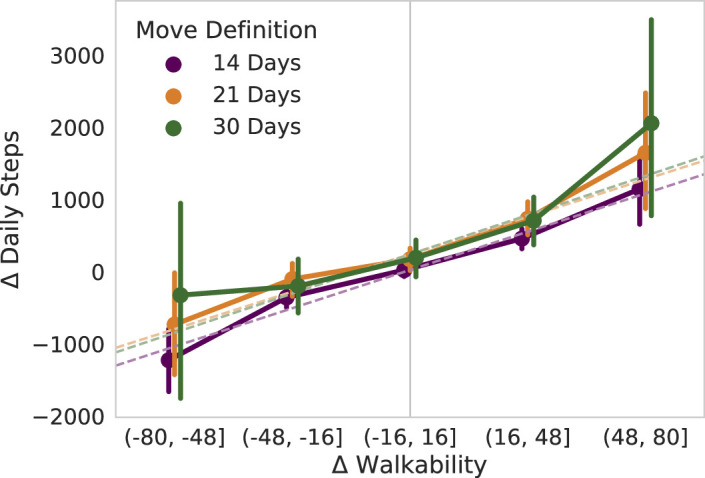
Different definitions of relocation lead to highly consistent results. After relocation, participants are required to stay in the new location for at least 14, 21, or 30 days. We find that all of these definitions lead to highly consistent results as most relocating participants stay for substantially longer periods of time (median 81 days). In the rest of the paper, we use the 14 day definition (purple).

**Extended Data Fig. 7:**
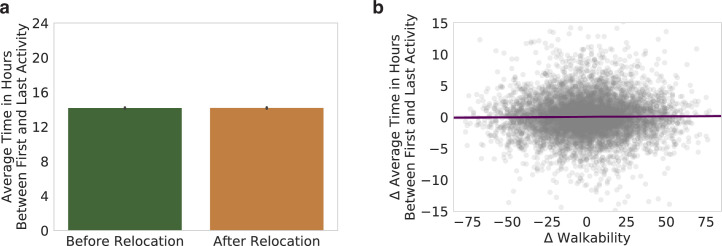
Higher physical activity in more walkability cities is not explained by differences in estimated wear time. Participants have an average span of 14.2h between the first and last recorded step, our proxy for daily wear time (Methods). **a**, Wear time estimates before and after relocation are 14.16 hours and 14.18 hours, respectively, with no significant difference (*P* = 0.807; t-test). **b**, We find no significant association between relocation-induced difference in walkability and wear time. The line shows the best linear fit using data from all relocations. Its slope is not significantly different from zero (slope 0.0014; *P* = 0.371; t-test). These results suggest that differences in recorded steps after relocation are due to actual differences in physical activity behavior and are not explained by differences in wear time.

While relocation uniquely enabled the quasi-experimental study of behavioural changes in different environments, there may be selection effects driving relocation, often referred to as residential self-selection. According to a 2013 US Census Bureau report, 98% of people moved primarily for reasons of housing, family and employment^85^. Less than 1% of people moved primarily for health reasons. There are some categories that might, in part, include people who want to reduce their dependence on cars. These include ‘health reasons’ (0.4%), ‘other housing-related’ (14.0%), ‘wanted better neighborhood/less crime’ (3.2%) and ‘to be closer to work/easier commute’ (5.4%), suggesting that at least 77% of participants moved for reasons completely unrelated to car dependence^85^. In addition, neighbourhood selection may be influenced by personal preferences such as exercise and walking activities^20^. With respect to this possibility, note that we found no indication of increases in physical activity after moving to a location of similar walkability (Figs. 2a and 3c). This suggests that those relocating participants are not simply more motivated to exercise, on average, but that changes in physical activity may be explained by the changing built environment. It is possible that selection effects were absent because participants may not have perceived themselves as being observed, in contrast to previous studies that featured explicit, short periods of monitoring (Hawthorne effect). We further acknowledge that other city characteristics may affect walking and be correlated with the city’s walkability (for example, length of work days). We investigated potential selection effects further by comparing the population of relocating mobile app users, first, to the overall US population, and, second, to the overall mobile app user population, including non-relocating app users. We found that the relocating participant population is similar in age (36 versus 37.7 years median age) and gender (49.8% versus 51.0% female, *P* = 0.132; Student’s *t*-test) to the US population (Extended Data Fig. 3). We adjusted for differences in age for the simulation estimates in Fig. 3f and Extended Data Fig. 1. Within the app user population, we found that movers and non-movers (that is, relocating and non-relocating participants) tend to be close in age (43.8 versus 37.9 and 38.5 versus 33.7 average age for men and women, respectively; Extended Data Fig. 4a,b), and weight status (68.1% versus 59.8% and 45.6% versus 44.3% overweight and obese for men and women, respectively; Extended Data Fig. 4c,d). However, movers were generally more physically active than non-movers (6,284 versus 5,825 and 5,279 versus 4,635 average daily steps for men and women, respectively; Extended Data Fig. 4e,f). Furthermore, we found that within movers, those that relocate to higher-, similar- and lower-walkability locations were similar in age, weight status and previous physical activity levels (Extended Data Fig. 5).

### Simulating the impact of walkability improvements

We simulated the impact of US nationwide walkability improvements on US population physical activity levels. Concretely, we simulated the impact of increasing US city walkability scores to a constant target walkability score between 1 and 100. We also highlight the walkability scores of Chicago and Philadelphia (78) as well as New York City (89) to aid interpretation. As the relocation population was not explicitly drawn to be representative of the US population, we adjusted our estimates through ratio-based post-stratification weights across age-based strata^86^. We used civilian population estimates from the US Census Bureau for 2016 as the target population distribution. While there were no significant differences in the gender distribution (49.8% female versus 51.0% female, *P* = 0.132; Extended Data Fig. 3a), we found slight differences in age (36.0 versus 37.7 years median age; Extended Data Fig. 3b), which we corrected for through sampling weights. We acknowledge that other selection effects and heterogeneous treatment effects may exist. Using a bootstrap with 1,000 replications, we estimated the difference in the overall US population that would meet US national aerobic physical activity guidelines for MVPA^1^ after relocating based on the relocation-induced difference in walkability. We used a linear regression model and data from relocations associated with both walkability increases and decreases. We estimated the total fraction of US population meeting aerobic physical activity guidelines as the sum between the fraction of people already meeting these guidelines before relocating plus the estimated addition based on the regression model. Confidence intervals represent bootstrapped 95% confidence intervals. Final estimates are depicted in Fig. 3f and Extended Data Fig. 1.

### Reporting summary

Further information on research design is available in the Nature Portfolio Reporting Summary linked to this article.

## Online content

Any methods, additional references, Nature Portfolio reporting summaries, source data, extended data, supplementary information, acknowledgements, peer review information; details of author contributions and competing interests; and statements of data and code availability are available at 10.1038/s41586-025-09321-3.

## Supplementary information

Supplementary Information This file contains Supplementary Figs. 1–7.

Reporting Summary

## Figures and Tables

**Extended Data Table 1:**
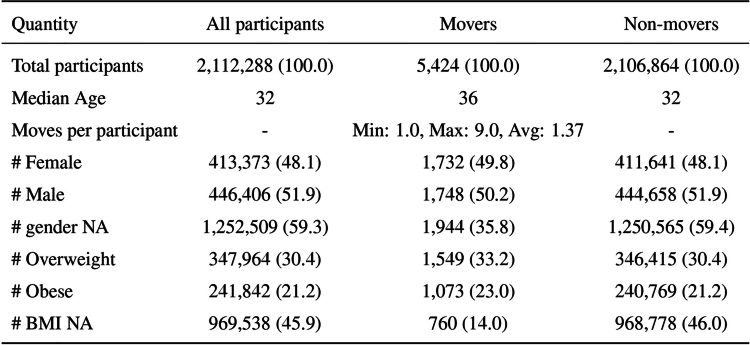
Summary of demographic statistics for the study participants (Methods)

Study observation period ranged from March 2013 to February 2016. Percentages are in parentheses. NA refers to missingness in data.

**Extended Data Table 2:**
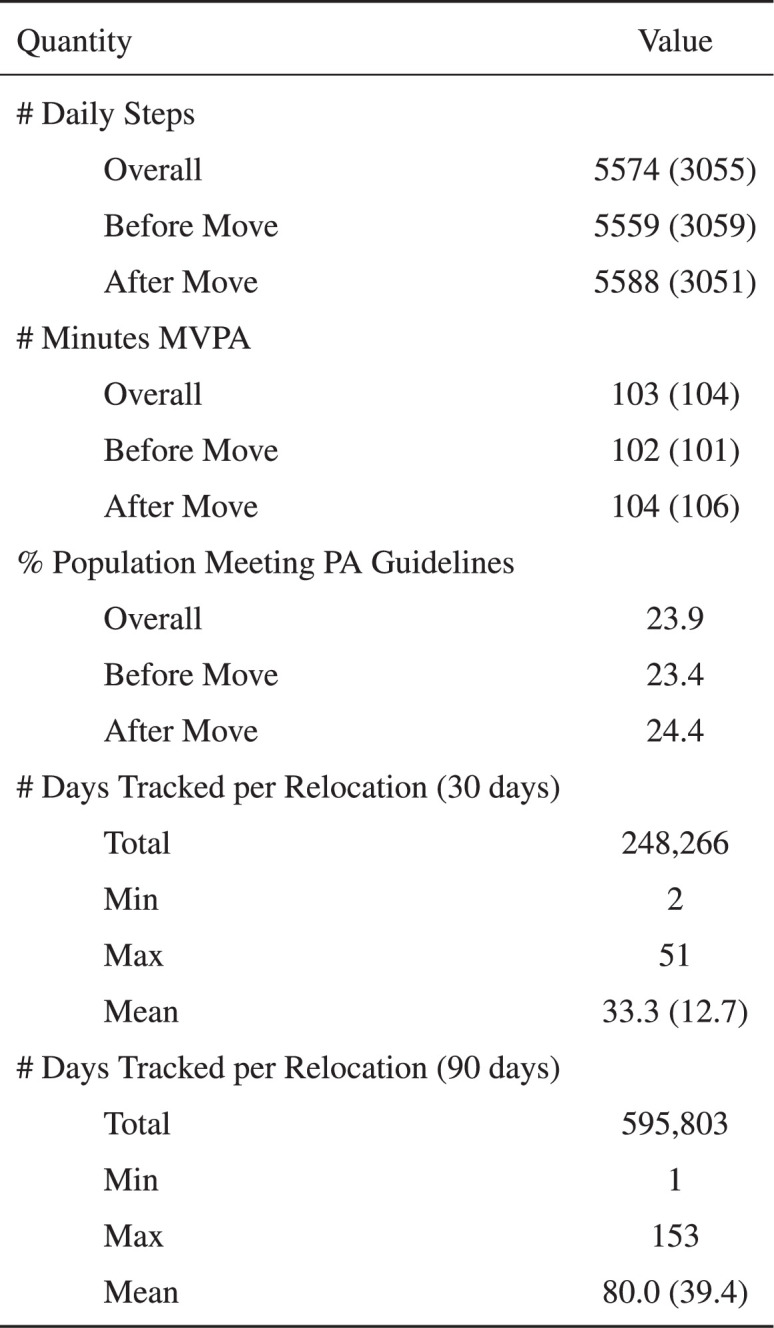
Summary of physical activity statistics for the study participants (Methods)

Statistics are averages unless otherwise noted with standard deviations in parentheses.

**Extended Data Table 3:**
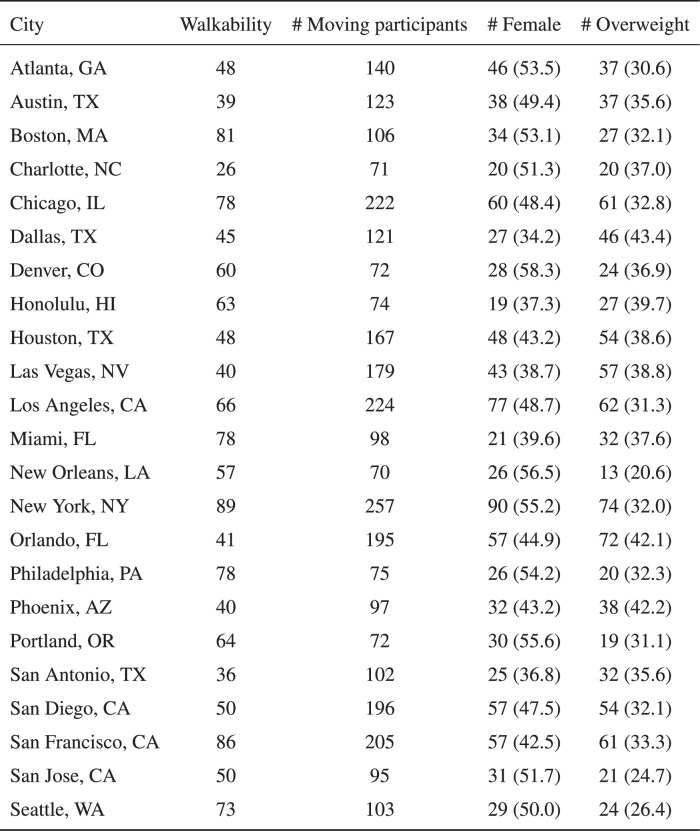
Location and walkability statistics for all locations included in our study with at least 70 moving participants (sorted alphabetically)

Percentages are in parentheses.

## Data Availability

Data are available at GitHub (https://github.com/behavioral-data/movers-public).

## Code availability

Code is available at GitHub (https://github.com/behavioral-data/movers-public).
