# Supplementary material for: Countrywide natural experiment links built environment to physical activity
Source: Nature. 2025 Aug 13;645(8080):407–13. doi: 10.1038/s41586-025-09321-3 (PMC12422959; doi:10.1038/s41586-025-09321-3)
Supplement: Supplementary file 1 — This file contains Supplementary Figs. 1–7. [file 41586_2025_9321_MOESM1_ESM.pdf]

nature portfolio

https://doi.org/10.1038/s41586-025-09321-3

Supplementary information

Countrywide natural experiment links built environment to physical activity

In the format provided by the authors and unedited

Supplementary Information

This Supplementary Information includes in this single file:

- Supplementary Figure S1: Participants' physical activity levels undergo significant changes following relocation to and from specific locations of different walkability.
- Supplementary Figure S2: Changes in average daily steps following relocation between specific walkability score quintiles.
- Supplementary Figure S3: Relocations are not uniformly distributed across the year and activity levels need to be adjusted to exclude seasonal effects (Methods).
- Supplementary Figure S4: Five days immediately before and after relocation are filtered out to exclude effects from the relocation process itself.
- Supplementary Figure S5: Relationship between city walkability and physical activity holds for relocations to the same Köppen climate type.
- Supplementary Figure S6: Relationship between city walkability and physical activity holds for relocations within any given season.
- Supplementary Figure S7: Relationship between city walkability and physical activity holds within U.S. cities of similar income.

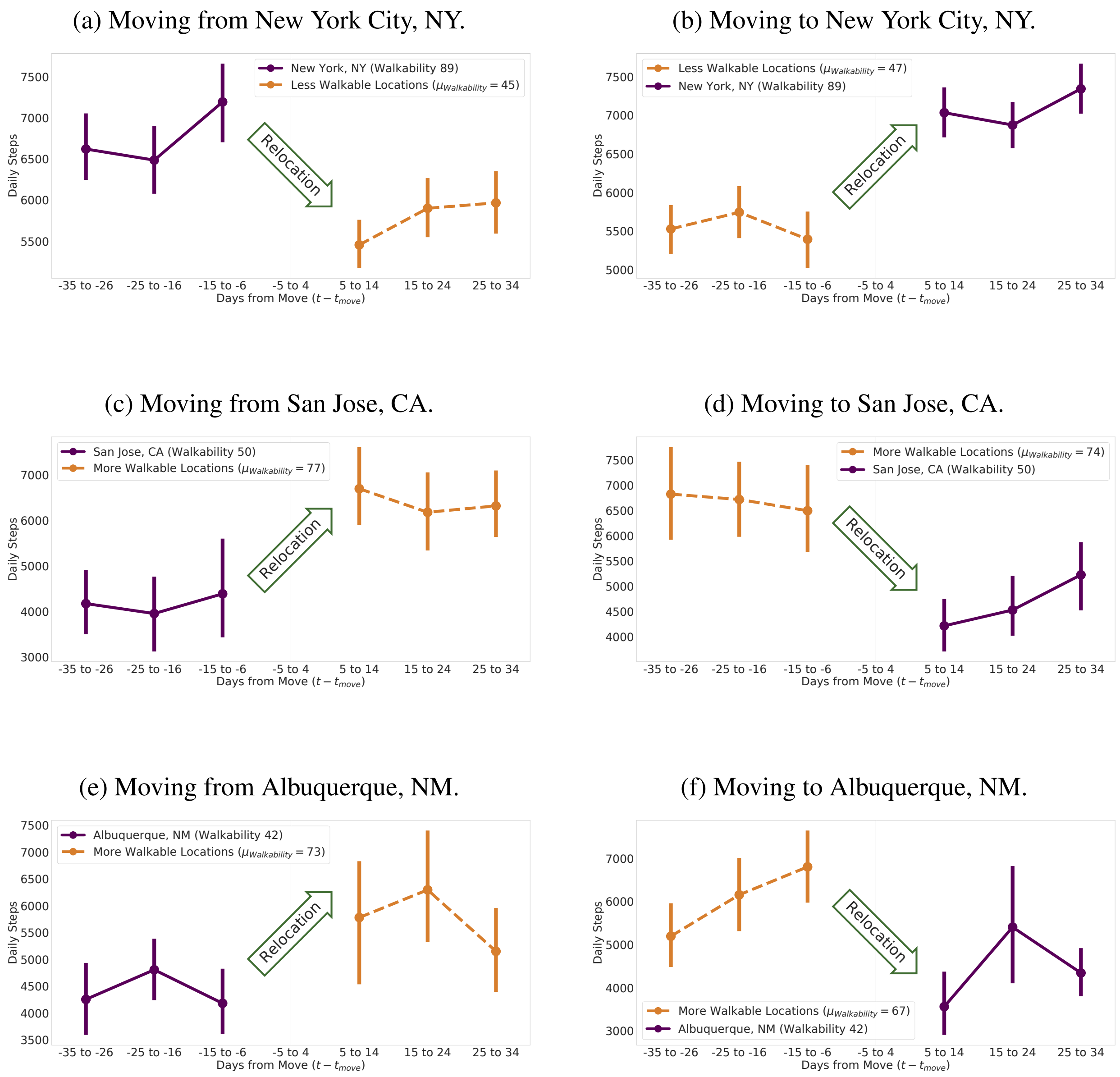

Supplementary Figure S1: **Participants' physical activity levels undergo significant changes following relocation to and from specific locations of different walkability.** Examples show physical activity levels for participants moving from/to New York, NY, San Jose, CA, and Albuquerque, NM (differences in walkscore of more than one standard deviation of 15.4 points). Physical activity levels change significantly by about 1,200 - 1,400 daily steps depending on the location. Note the symmetry between moving from (left) and to (right) specific locations. All error bars throughout these Supplementary Materials correspond to bootstrapped 95% confidence intervals.

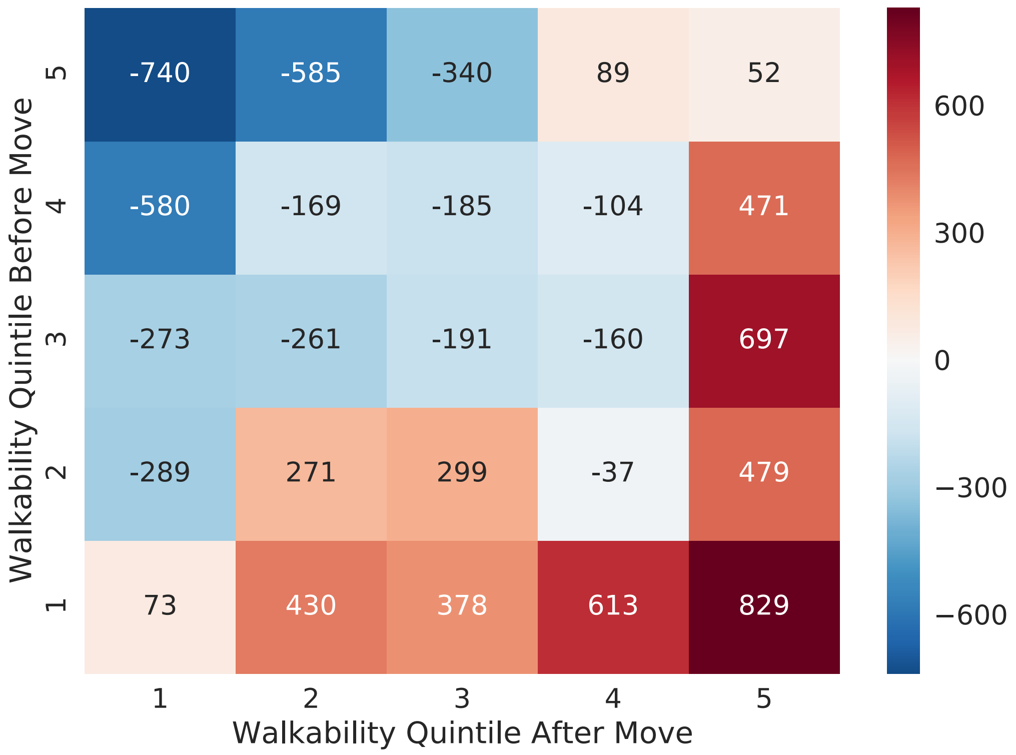

Supplementary Figure S2: **Changes in average daily steps following relocation between specific walkability score quintiles.** Changes in physical activity levels are approximately symmetric and close to zero for relocations to the same walkability score quintile.

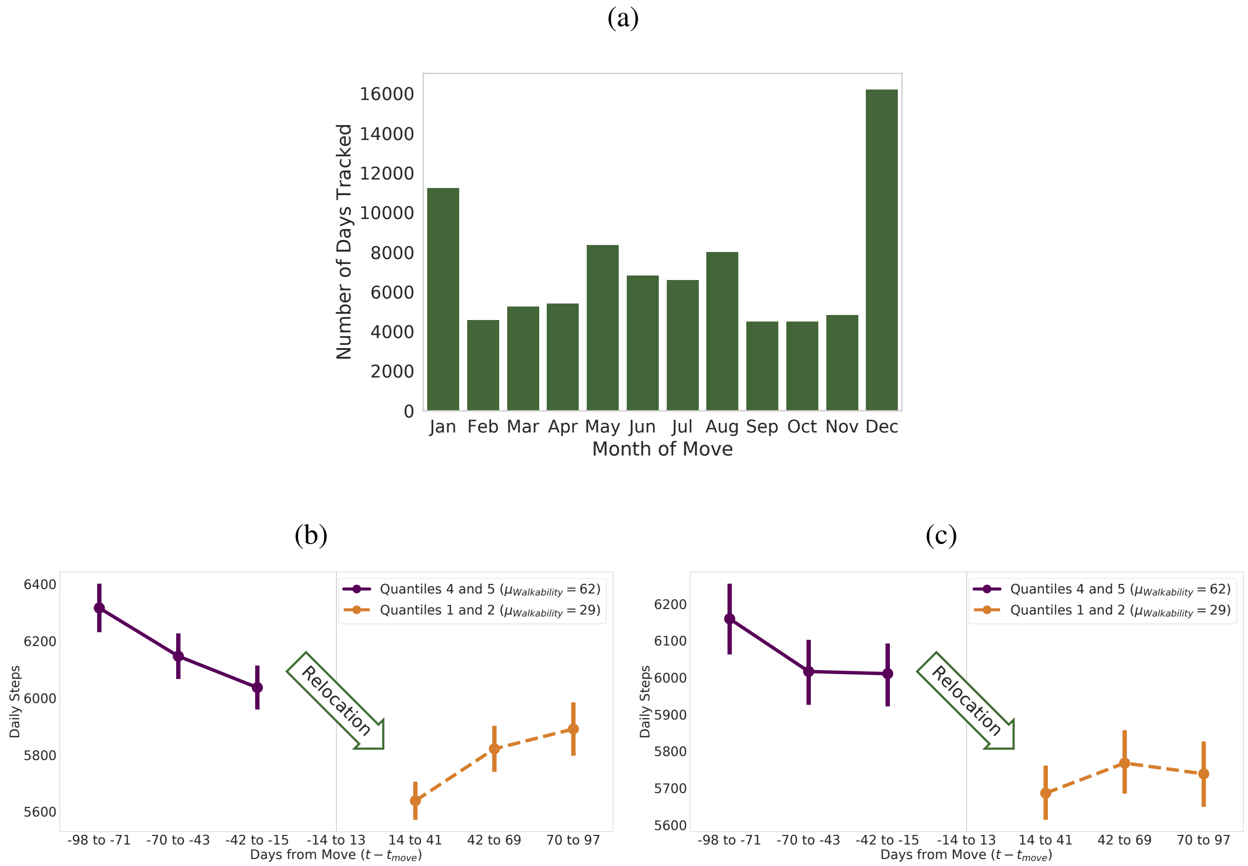

Supplementary Figure S3: **Relocations are not uniformly distributed across the year and activity levels need to be adjusted to exclude seasonal effects (Methods). a**, Histogram of number of days with tracked physical activity is non-uniform with more relocations in December and January. **b**, Changes in physical activity when relocating from highly walkable to less walkable built environments, without adjusting for the number of relocations across seasons. We observe clearly changing physical activity levels before and after relocation that may be explained by the more moderate weather in fall and spring versus winter. **c**, After adjusting for seasonal effects by weighting all months equally, physical activity levels before and after relocation are more stable.

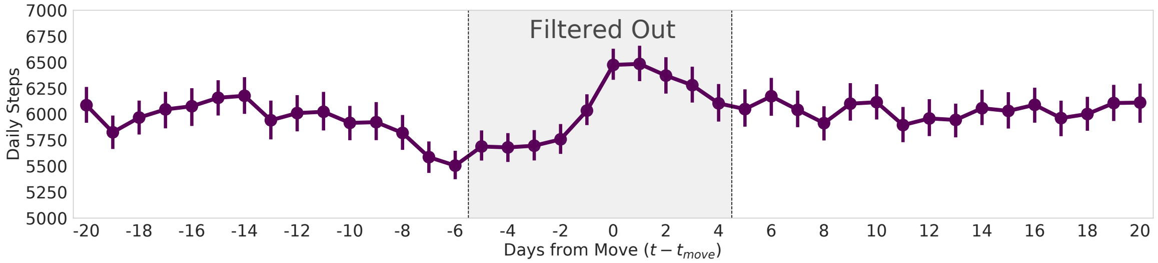

Supplementary Figure S4: **Five days immediately before and after relocation are filtered out to exclude effects from the relocation process itself.** Outside this interval, participants' physical activity levels were relatively stable. Figure depicts relocations to similarly walkable locations (walkability score difference between -16 and 16).

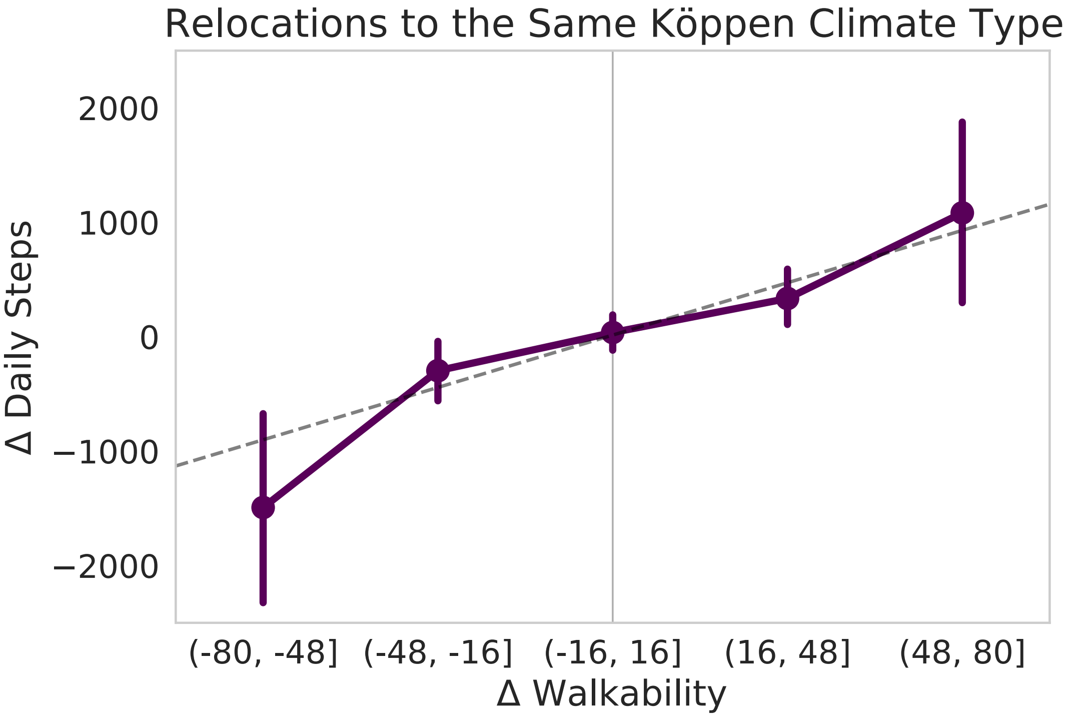

Supplementary Figure S5: **Relationship between city walkability and physical activity holds for relocations to the same Köppen climate type.** We find that that relocations to more walkable cities are associated with significant increases in physical activity across moves to the same Köppen climate type. These results suggest that our main result—city walkability impacts physical activity—is independent of any potential climate bias in our sample (i.e., moves to a more favorable climate for physical activity are not driving the differences we observe).

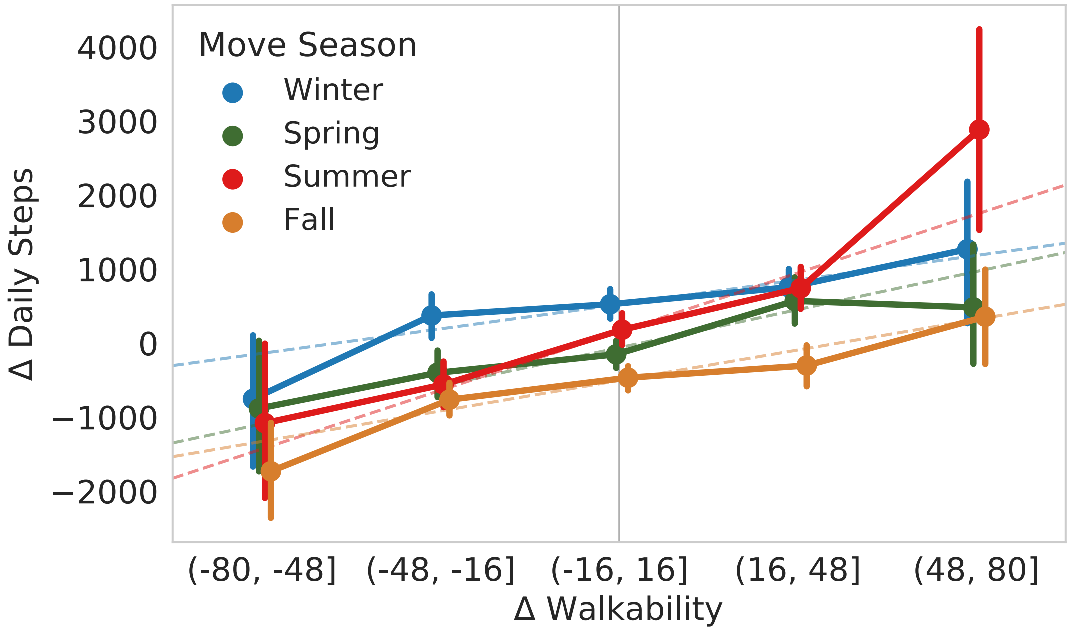

Supplementary Figure S6: **Relationship between city walkability and physical activity holds for relocations within any given season.** We find that that relocations to more walkable cities are associated with significant increases in physical activity across moves that occur within any given season (all $P < 10^{-3}$; t-test). These results suggest that our main result—city walkability impacts physical activity—is independent of any potential temporal bias in our sample (i.e., moves during a time that is more favorable for physical activity are not driving the differences we observe).

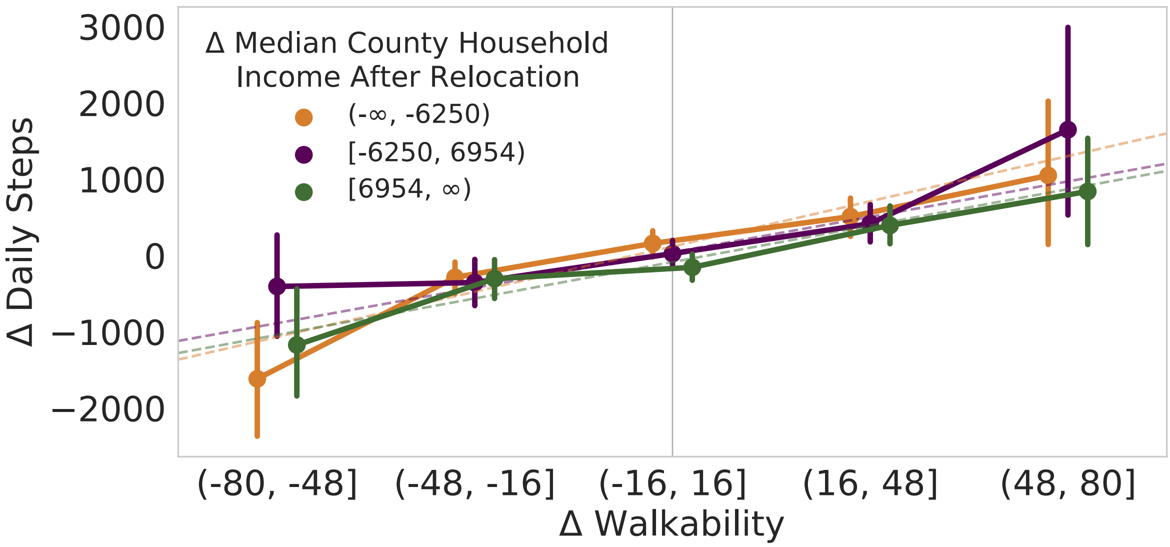

Supplementary Figure S7: **Relationship between city walkability and physical activity holds within U.S. cities of similar income.** We find that that relocations to more walkable cities are associated with significant increases in physical activity across all three groups (increasing, similar, and decreasing median county household income in USD). These results suggest that our main result—city walkability impacts physical activity—is robust to potential socioeconomic bias in our sample.
