# Supplementary material for: Countrywide natural experiment links built environment to physical activity
Source: Nature. 2025 Aug 13;645(8080):407–13. doi: 10.1038/s41586-025-09321-3 (PMC12422959; doi:10.1038/s41586-025-09321-3)
Supplement: Supplementary file 2 — Reporting Summary [file 41586_2025_9321_MOESM2_ESM.pdf]

nature portfolio

Corresponding author(s): Tim Althoff

Last updated by author(s): 3/31/2025

Reporting Summary

Nature Portfolio wishes to improve the reproducibility of the work that we publish. This form provides structure for consistency and transparency in reporting. For further information on Nature Portfolio policies, see our Editorial Policies and the Editorial Policy Checklist.

Statistics

For all statistical analyses, confirm that the following items are present in the figure legend, table legend, main text, or Methods section.

n/a	Confirmed	
☐	☒	The exact sample size (*n*) for each experimental group/condition, given as a discrete number and unit of measurement
☐	☒	A statement on whether measurements were taken from distinct samples or whether the same sample was measured repeatedly
☐	☒	The statistical test(s) used AND whether they are one- or two-sided<br>*Only common tests should be described solely by name; describe more complex techniques in the Methods section.*
☐	☒	A description of all covariates tested
☐	☒	A description of any assumptions or corrections, such as tests of normality and adjustment for multiple comparisons
☐	☒	A full description of the statistical parameters including central tendency (e.g. means) or other basic estimates (e.g. regression coefficient) AND variation (e.g. standard deviation) or associated estimates of uncertainty (e.g. confidence intervals)
☐	☒	For null hypothesis testing, the test statistic (e.g. *F*, *t*, *r*) with confidence intervals, effect sizes, degrees of freedom and *P* value noted<br>*Give P values as exact values whenever suitable.*
☒	☐	For Bayesian analysis, information on the choice of priors and Markov chain Monte Carlo settings
☒	☐	For hierarchical and complex designs, identification of the appropriate level for tests and full reporting of outcomes
☒	☐	Estimates of effect sizes (e.g. Cohen's *d*, Pearson's *r*), indicating how they were calculated

Our web collection on statistics for biologists contains articles on many of the points above.

Software and code

Policy information about availability of computer code

Data collection	Azumio Argus smartphone app
Data analysis	Analysis data and code are available at https://github.com/behavioral-data/movers-public<br>Software include Python with packages numpy, scipy, pandas, matplotlib, seaborn

For manuscripts utilizing custom algorithms or software that are central to the research but not yet described in published literature, software must be made available to editors and reviewers. We strongly encourage code deposition in a community repository (e.g. GitHub). See the Nature Portfolio guidelines for submitting code & software for further information.

Data

Policy information about availability of data

All manuscripts must include a data availability statement. This statement should provide the following information, where applicable:

- Accession codes, unique identifiers, or web links for publicly available datasets
- A description of any restrictions on data availability
- For clinical datasets or third party data, please ensure that the statement adheres to our policy

Data was collected from Azumio Argus smartphone app that allows individuals to track activities including physical activity, and Walkscore.com. All aggregated data necessary for reproducing results are available at https://github.com/behavioral-data/movers-public

Research involving human participants, their data, or biological material

Policy information about studies with human participants or human data. See also policy information about sex, gender (identity/presentation), and sexual orientation and race, ethnicity and racism.

Reporting on sex and gender	Research subjects self-reported gender information in the Azumio Argus app (optional).
Reporting on race, ethnicity, or other socially relevant groupings	Race, ethnicity were not collected and/or used in this study
Population characteristics	Refer to manuscript Tables 1,2,3
Recruitment	Research subjects were users of the Azumio Argus smartphone app, a free application for tracking activities. We analyzed anonymized, retrospective data collected during a 3-year observation period between 2013 and 2016 that were aggregated to city level or demographic categories. For details on data inclusion criteria, refer to manuscript Methods "Study Design", "Identifying Subject Relocation", and "Physical Activity Measure".
Ethics oversight	Stanford University IRB

Note that full information on the approval of the study protocol must also be provided in the manuscript.

Field-specific reporting

Please select the one below that is the best fit for your research. If you are not sure, read the appropriate sections before making your selection.

☐ Life sciences ☒ Behavioural & social sciences ☐ Ecological, evolutionary & environmental sciences

For a reference copy of the document with all sections, see nature.com/documents/nr-reporting-summary-flat.pdf

Behavioural & social sciences study design

All studies must disclose on these points even when the disclosure is negative.

Study description	We conducted a countrywide, prospective, longitudinal physical activity study of United States residents that evaluated their physical activity levels within the context of the walkability of their built environments before and after relocation. We leveraged the naturally occurring physical activity data that was captured by a health app on subjects' phones to compare each person's physical activity levels before and after they relocated to a different U.S. area.
Research sample	Participants were users of the Azumio Argus smartphone application. All data was anonymized and aggregated at various levels. Sample was a non-representative sample; for comparison with U.S. population of relocating people and the overall Azumio Argus user population, see our manuscript. For research sample details, refer to manuscript Methods "Study Design", "Identifying Subject Relocation", and "Physical Activity Measure".
Sampling strategy	Retrospective observational study, where sample size was determined by the size of the shared sample from Azumio. The manuscript investigates potential selection effects and determines that the size and nature of this data is sufficient for all highlighted statistical comparisons.
Data collection	Data was collected from Azumio Argus smartphone app activity tracking) and from Walkscore.com. All data was anonymized and aggregated at various levels.
Timing	March 2013 to February 2016
Data exclusions	We required subjects to have used the app to track their physical activity for at least 10 days within 30 days before and after their relocation (following previous work in https://www.nature.com/articles/nature23018). Subjects were excluded from a particular analysis if necessary information was unreported (for example, subjects with no reported age were excluded from the analysis of Figure 2b).
Non-participation	NA
Randomization	Subjects were not randomized to conditions (i.e., relocation). For a comparison between relocating and non-relocating users of the Azumio smartphone app, see Supplementary Figure 8.

Reporting for specific materials, systems and methods

We require information from authors about some types of materials, experimental systems and methods used in many studies. Here, indicate whether each material, system or method listed is relevant to your study. If you are not sure if a list item applies to your research, read the appropriate section before selecting a response.

Materials & experimental systems

n/a	Involved in the study
☒	☐ Antibodies
☒	☐ Eukaryotic cell lines
☒	☐ Palaeontology and archaeology
☒	☐ Animals and other organisms
☒	☐ Clinical data
☒	☐ Dual use research of concern
☒	☐ Plants

Methods

n/a	Involved in the study
☒	☐ ChIP-seq
☒	☐ Flow cytometry
☒	☐ MRI-based neuroimaging

Plants

Seed stocks	N/A
Novel plant genotypes	N/A
Authentication	N/A
